# Supplementary material for: Quantitative characterization of microstructure and research on spatial variation characteristics of loess of different strata in Luochuan, Shaanxi, China
Source: PLoS One. 2024 May 10;19(5):e0303089. doi: 10.1371/journal.pone.0303089 (PMC11086893; doi:10.1371/journal.pone.0303089)
Supplement: Attachment — Submitted filename: Response to Reviewers.docx [file pone.0303089.s002.docx]

Thank you to Reviewer#1 for their valuable review suggestions, and we will respond to each of the questions raised by Reviewer#1 here.

1) The Abstract should incorporate more quantitative data to provide a concise summary of the study's key findings.

Response: Thank you for the reviewer's suggestions. We have simplified and summarized the abstract. 2) It is advisable to include recommendations and potential future research directions in the Introduction to give readers a broader context.

Response: In the introduction section, We added research directions on loess hotspots, such as modified loess new materials, loess permeability and microstructure relationship.

3) The manuscript contains a few spelling errors; the authors are kindly requested to proofread and correct them.

Response: We have checked and corrected some spelling errors word by word and sentence by sentence throughout the entire text.

4) To strengthen the study's credibility, the authors should address error quantification and validation. This can be achieved by providing replication evidence, statistical interpretations, and confidence intervals for each data point. Ensuring that figures include error bars will help readers assess statistical significance between samples. Additionally, discussing data uncertainty and conducting appropriate statistical analyses to establish sample distinctions is essential.

Response: We added 95% confidence intervals and 95% prediction intervals for data statistics starting from the data, and reflected this information in the graph, such as Fig4(a), Fig5(a), Fig6(a), Fig8(a), Fig9(a), and Fig10(a).

5) The Conclusion section should be concise yet retain its clarity and relevance to readers. The authors should clearly articulate the novel and significant conclusions derived from their research.

Response: We have extracted and summarized the conclusion, and provided a clear and concise expression of the research results.

Thank you to Reviewer#2 for their valuable review suggestions, and we will respond to each of the questions raised by Reviewer#2 here.

1.The reviewer believes that the literature review in the introduction section lacks coverage of the latest research and recommends that the author add a description of the latest progress.

Assessing unsaturated permeability of loess under multiple rainfalls. Engineering Geology 324:107280.

Mechanical properties, microstructural evolution, and environmental impacts of recycled polypropylene fiber stabilized loess. Construction and Building Materials 400:132850.

The impact of paleoclimatic on the structural strength of loess paleosol sequences and its implications for tillage on the Loess Plateau: A case study from Luochuan profile. Soil and Tillage Research 236:105939

An experimental study on the behavior of loess-clay mixtures upon wetting and its implications for tillage on the Loess Plateau. Soil and Tillage Research 240:106071

Response: According to the reviewer#2's suggestion, we have added the latest research findings in the introduction section and cited the article recommended by the reviewer#2.

2. Experimental Method

The reviewer believes that the explanation of the experimental method for loess microstructure is not sufficient, and suggests that the author provide additional information.

Response: Based on the reviewer#2's suggestions, we have supplemented and optimized the experimental method to make it more specific.

3.The reviewer suggests that the article supplement or cite the geographical location and stratigraphic profile map of Luochuan, China.

Response: Based on the reviewer#2's suggestions, we have added relevant drawings about the Luochuan section, such as Fig 1.

4.The reviewer in the article believes that there are spelling errors in some words, and suggests that the author carefully check the correctness of the word writing in the article.

Response: Based on the reviewer#2's suggestions, we have checked and corrected some spelling errors word for word throughout the paper.

5.The reviewer believes that the stratigraphic identification number in Table .1 should be consistent with the stratigraphic identification number in the main text of the article, or adopt industry recognized writing standards uniformly.

Response: Based on the reviewer#2's suggestions, we have unified the expression of stratigraphic numbering throughout the paper.

6.The reviewer suggests that the author should carefully check the article format in accordance with the requirements of the journal.

Response: We have thoroughly checked the articles according to the journal's requirements to ensure they meet the journal's formatting requirements.
